# Complement is activated in progressive multiple sclerosis cortical grey matter lesions

**DOI:** 10.1186/s12974-016-0611-x

**Published:** 2016-06-22

**Authors:** Lewis M. Watkins, James W. Neal, Sam Loveless, Iliana Michailidou, Valeria Ramaglia, Mark I. Rees, Richard Reynolds, Neil P. Robertson, B. Paul Morgan, Owain W. Howell

**Affiliations:** Institute of Life Sciences, Swansea University School of Medicine, Swansea, SA2 8PP UK; Institute of Infection and Immunity, Cardiff University, Cardiff, UK; Institute of Psychological Medicine and Clinical Neuroscience, Cardiff University, Cardiff, UK; Department of Genome Analysis, Academic Medical Centre, Amsterdam, The Netherlands; Division of Brain Sciences, Imperial College London, London, UK

**Keywords:** Complement, Grey matter lesion, Innate immunity, Multiple sclerosis, Neurodegeneration

## Abstract

**Background:**

The symptoms of multiple sclerosis (MS) are caused by damage to myelin and nerve cells in the brain and spinal cord. Inflammation is tightly linked with neurodegeneration, and it is the accumulation of neurodegeneration that underlies increasing neurological disability in progressive MS. Determining pathological mechanisms at play in MS grey matter is therefore a key to our understanding of disease progression.

**Methods:**

We analysed complement expression and activation by immunocytochemistry and in situ hybridisation in frozen or formalin-fixed paraffin-embedded post-mortem tissue blocks from 22 progressive MS cases and made comparisons to inflammatory central nervous system disease and non-neurological disease controls.

**Results:**

Expression of the transcript for *C1qA* was noted in neurons and the activation fragment and opsonin C3b-labelled neurons and glia in the MS cortical and deep grey matter. The density of immunostained cells positive for the classical complement pathway protein C1q and the alternative complement pathway activation fragment Bb was significantly increased in cortical grey matter lesions in comparison to control grey matter. The number of cells immunostained for the membrane attack complex was elevated in cortical lesions, indicating complement activation to completion. The numbers of classical (C1-inhibitor) and alternative (factor H) pathway regulator-positive cells were unchanged between MS and controls, whilst complement anaphylatoxin receptor-bearing microglia in the MS cortex were found closely apposed to cortical neurons. Complement immunopositive neurons displayed an altered nuclear morphology, indicative of cell stress/damage, supporting our finding of significant neurodegeneration in cortical grey matter lesions.

**Conclusions:**

Complement is activated in the MS cortical grey matter lesions in areas of elevated numbers of complement receptor-positive microglia and suggests that complement over-activation may contribute to the worsening pathology that underlies the irreversible progression of MS.

**Electronic supplementary material:**

The online version of this article (doi:10.1186/s12974-016-0611-x) contains supplementary material, which is available to authorized users.

## Background

Multiple sclerosis (MS) is an inflammatory, demyelinating and neurodegenerative disease of young adults. Damage can occur throughout the central nervous system, and the pathology of the grey matter can be extensive [1, 2]. Progressive MS, marked by increasing irreversible disability and reduced quality of life, is characterised pathologically by extensive cortical demyelination [3]. Magnetic resonance imaging has demonstrated that an increasing number of cortical lesions, and lesions of deep grey matter, are predictive of disease course [4]. Grey matter pathology is seen from the earliest stages, and inflammation is linked to neurodegeneration throughout the disease [5]. There are now concerted efforts to better understand the innate and adaptive immune mechanisms that drive this pathology to identify disease relevant prognostic markers and new therapeutic opportunities.

The complement system is central to innate and adaptive immune responses. Complement is synthesised in the brain, and adult human neurons are a major source of parenchymal complement [6], which can also be generated systemically. Complement is important for synaptic pruning during development, complement signalling causes neuronal morphological changes in the adult and complement-labelled neurons are targeted by complement receptor-bearing phagocytes [7–10]. Complement is activated through the classical, lectin and alternative pathways that generate anaphylatoxins C3a and C5a and opsonins, including C3b [11, 12]. Build-up of complement fragment C3b can lead to C5 convertase formation with proteolysis of the C5 component and subsequent activation of the terminal pathway leading to the formation of the membrane attack complex (MAC). To avoid self-injury, host cells express an array of complement regulatory proteins (Cregs) that, for example, inhibit C3-cleaving enzymes (factor H), prevent C1q assembly with C1r, s and initiation of the classical pathway (C1-inhibitor) or block assembly of the MAC (clusterin) [13]. Intrathecal and blood-borne levels of complement proteins mirror the MS disease profile [14–17], but we need to know more about the role of complement in pathogenesis.

Evidence for complement activation in MS grey matter is mixed, with some reporting little evidence for complement activation in purely cortical lesions [18]. Others have shown complement to be differentially expressed [19–21] and the complement recognition and initiation protein C1q to be associated with degenerating synapses in the MS hippocampus [22]. As yet, Creg expression in MS grey matter has not been reported. We have examined the localisation of complement recognition molecules (C1q), activation products (C3b, Bb, MAC), regulators (factor H, C1 inhibitor, clusterin) and receptors (C3aR, C5aR and complement receptor 3/ CD11b) for the first time in order to better understand the immune mechanisms of MS cortical grey matter pathology relevant to disease progression.

## Methods

### Study cohort

Tissue was provided by the UK Multiple Sclerosis Society Tissue Bank (MSSTB) at Imperial College (MS and non-neurological controls, including cryopreserved samples) and the Oxford Brain Bank, Oxford University (inflammatory and non-neurological controls) with appropriate research ethics approval (South West Wales REC study approval 13/WA/0292). Of these samples, 22 cases of neuropathologically confirmed MS, 15 non-neurological controls and eight non-MS neurological controls with glial activation and neuroinflammation (four ischaemic stroke, four viral encephalitis; referred to collectively as “non-MS inflammatory controls”) were used for the main data collection (Table 1 and Additional file 1:Table S1). Non-neurological and non-MS inflammatory controls were selected based on the availability of tissue blocks from the pre-determined study regions and gender and age similar to the available MS cohort. In all instances, the fresh brain was cut into 1-cm-thick coronal slabs before either cryopreserving in cold isopentane on a bed of dry ice or placed in freshly prepared fixative (for formalin-fixed, paraffin-embedded samples). Sections from representative anatomically matched brain regions were prepared for each case as follows: (1) superior frontal gyrus, (2) cingulate gyrus, (3) thalamus, and (4) hippocampus and inferior temporal lobe. Cryosections from region-matched blocks of frontal, cingulate and hippocampus and inferior temporal lobe were available for the complete MS cohort and, together with an additional eight non-neurological controls (MSSTB), were used to assess complement terminal pathway activation (staining for C9neo and clusterin) and complement receptor staining.

**Table 1:** Summary of multiple sclerosis and control groups used for quantitative analysis

Cohort	Status (*n*)	Gender	Age at death	Disease duration	PMD
Multiple sclerosis	18 SPMS	9M:13F	50 years (38–66)	25 years (10–39)	17 h (9–26)
	4 PPMS				
Inflammatory Ctrls	4 viral encephalitis	6M:2F	37 years (17–65)	n/a	36 h (24–72)
	4 ischaemia				
Non-neurological Ctrls	15 controls	9M:6F	67 years (35–88)	n/a	24 h (5–48)

Inflammatory controls are non-MS neurological controls characterised by neuroinflammation and gliosis. Group values showed as median (data range). See (Additional file 1: Table S1) for individual case details including cause of death, disease activity at death and sampled blocks

*Ctrls* controls, *disease duration* determined retrospectively from time of first symptom onset to death, *F* female, *M* male, *PMD* post-mortem delay, *PPMS* primary progressive MS, *SPMS* secondary progressive MS, *n/a* not applicable

### Tissue characterisation

**Fig. 1:**
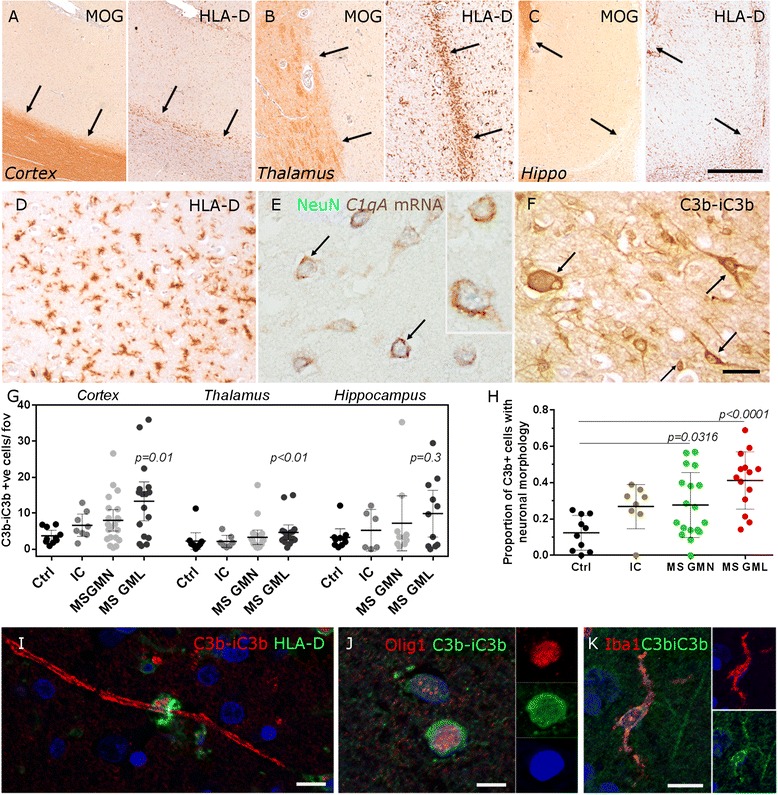
Complement activation in MS grey matter lesions. Complement expression was investigated in cortical (**a**) and subcortical (**b**, **c**) grey matter characterised by anti-myelin oligodendrocyte glycoprotein (*MOG*) and anti-HLA-D immunochemistry, to reveal demyelinated lesions (lesion edge marked by *arrows* in **a–c**) and activated microglia, in the absence of amoeboid macrophages (**d**). Complement *C1qA* mRNA (*red-brown* reaction product) was expressed in MS cortical grey matter neurons (*NeuN+*, *blue*; **e**), whilst complement activation fragment C3b-iC3b (hereafter referred to as C3b+) was localised to the membrane and cytoplasm of neurons and glia (cell-associated immunolabelling noted by *arrows*, **f**). Quantitative analysis of the density of C3b+ cells in cortical, deep grey matter and hippocampus from MS, non-MS inflammatory control and non-neurological controls (**g**). C3b+ cell density was increased in cortical and deep grey matter lesions in comparison to area-matched non-neurological controls (**g**). The proportion of C3b+ cells with a neuronal morphology was increased in MS cortex in comparison to non-neurological control (**h**). Anti-C3b immunolabelled myelin closely associated with a HLA+ macrophage (**i**), oligodendrocytes (**j**) and microglia (**k**). *Abbreviations*: *Ctrl* non-neurological control cohort, *IC* non-MS inflammatory controls, *GMN* grey matter normal, *GML* grey matter lesion. Each data point represents the mean value per area of interest (lesion, normal-appearing or control) for the respective grey matter field, per case. Group means and 95 % confidence interval are plotted and compared by Kruskal-Wallis and Dunn’s multiple comparison post-test. *Scale bars*: **a**–**c**, 2 mm; **d**–**f**, 50 μm; **i**–**k**, 5 μm

**Fig. 2:**
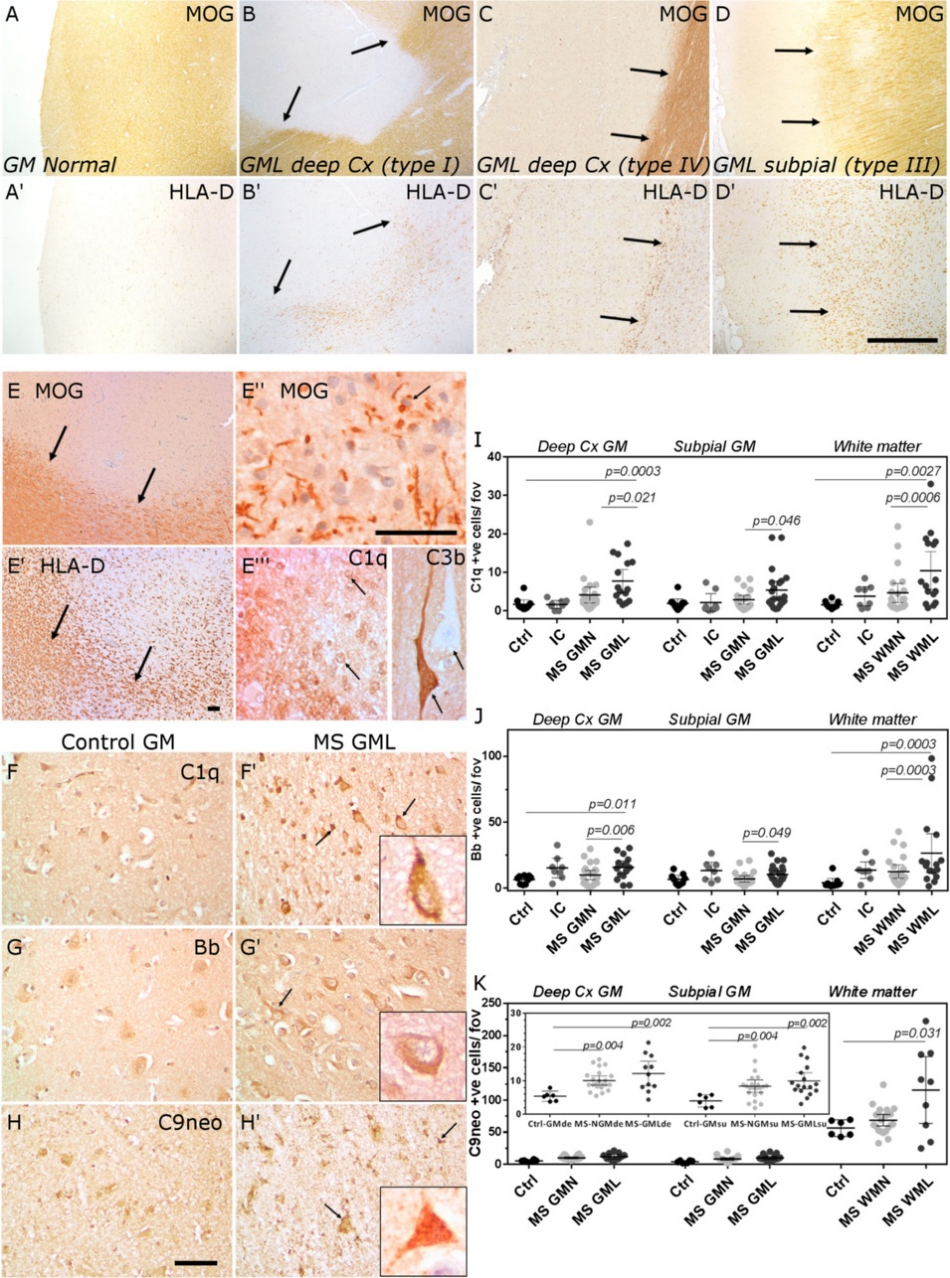
Classical, alternative and terminal complement pathway activation in MS cortical lesions. Control (**a**) and MS cortex (**b**–**d**) immunostained for MOG and HLA-D to reveal lesions affecting the deeper cortical laminae (**b**, **c**, *arrows* indicate lesion edge of a type I and type IV lesion, respectively) and a subpial lesion (**d**). Within an active cortical grey matter lesion (**e–e**′′ *arrows* indicate lesion edge in **e** and **e**′ and early myelin degradation products in **e**′′), C1q+ and C3b+ immunostaining was noted (*arrows* indicate labelled cells. Note the total absence of C3b immunolabelling of a neuron directly adjacent to a C3b+ cell (**e**′′). Immunolabelling of cells morphologically resembling neurons and glia in the deep cortical laminae of control and MS lesions indicated activation of the classical (C1q+, **f**, **f**′), alternative (fragment Bb+, **g**, **g**′) and terminal (C9neo+, **h**, **h**′) complement pathways (*arrows* in insets in **f**′–**h**′ highlight immunopositive neurons or glia). The numerical density of C1q+ (**i**), fragment Bb+ (**j**) and C9neo+ (**k**) cells was increased in grey matter and white matter lesions. Each data point represents the mean value per area of interest per case and group means and 95 % confidence intervals plotted. Groups were compared by Kruskal-Wallis and Dunn’s multiple comparison post-test. *Scale bars*: **a**–**d**, 2 mm; **e**–**g**, 100 μm

All cases were processed histologically for luxol fast blue/cresyl fast violet and haematoxylin/eosin, and subsequent sections were stained with antibodies to myelin-oligodendrocyte-glycoprotein (MOG) and human leukocyte antigen (HLA-D) to determine tissue architecture, cellular infiltration, demyelination and the density of microglia/macrophages (Figs. 1 and 2 and Additional file 1: Figure S1). White matter lesions (WML), deep grey matter (thalamus) and hippocampus lesions of MS were classified as follows: active (demyelinated lesion core confluent with HLA-D+ microglia/macrophages and the presence of early myelin (MOG+ inclusion) degradation products); chronic active (HLA-D+ microglia/macrophages restricted to the lesion edge and the presence of early and late (LFB+ inclusion) myelin degradation products) or chronic inactive (pale or no rim of ramified microglia (with late myelin degradation products) at the edge). Grey matter lesions (GML) of the frontal, cingulate and temporal gyri were characterised based on location within the cortical laminae and were described as subpial (type III), intracortical (type II) and leukocortical involving the subcortical white matter (type I) or as lesions spanning the entire width of cortex from the pia to the leukocortical junction, but without involving the white matter (type IV) [23]. Our analysis of lesions affecting the deep cortical laminae (layers V and VI) included both type I and IV lesion areas. All cortical GMLs used in this study (subpial and those affecting the deep cortical laminae) were characterised as chronic (active or inactive) based on the number and distribution of HLA-D+ microglia/macrophages and the presence of early (chronic active) or late (chronic inactive) myelin degradation products [24].

### Immunostaining protocols and image capturing

Paraffin wax sections were de-waxed, rehydrated and subjected to heat-induced epitope retrieval as described previously [25]. Following overnight incubation with the primary antibody, sections were incubated with biotinylated secondary antibody and avidin-biotin peroxidase complex with diaminobenzidine as the chromogen (Impact DAB; Vector Laboratories Ltd., Peterborough, Cambridgeshire, UK). Individual antibody details are listed in Additional file 1: Table S2. Snap-frozen, unfixed cryosections were air-dried, fixed in methanol or 4 % paraformaldehyde and quenched with H_2_O_2_ before immunostaining. Immunofluorescence staining was performed on wax or snap-frozen sections by sequential antibody incubation and detection, followed by diamidino-2-phenylindole (DAPI) counterstaining. In all instances, sections from each MS case for each antibody were immunostained in the same experimental run to ensure comparability of labelling. All experiments included primary antibody-negative controls and irrelevant species-specific antisera as positive controls. Sections were viewed on a Leica DRMB brightfield microscope (Leica Microsystems, Milton Keynes, Buckinghamshire, UK), a Zeiss Axio Imager under epifluorescence or a Zeiss LSM 710 confocal (Carl Zeiss Ltd., Cambridge, Cambridgeshire, UK).

### Quantitative analysis

All quantification and analysis was performed with the researcher blinded to the case identity. The mean number of immunostained cells was calculated for each complement protein or cell phenotypic marker of interest from ×100 images (field of view (fov) area = 0.3 mm^2^) of regions of interest: normal or demyelinated cortical laminae I–III (i.e. subpial lesions); normal or demyelinated cortical laminae V–VI (i.e. leukocortical and type IV lesions); normal or demyelinated subcortical white matter; and for C3b-iC3b only, normal or lesioned thalamus (ventral nucleus) and normal or lesioned CA1 of the rostral hippocampus. Positively stained cells were manually tagged in ImageJ (http://imagej.nih.gov/ij/) using the “multipoint” tool. Layer V NeuN-immunopositive neurons were automatically counted using the “analyse particles” tool following image transformation and thresholding. Changes in nuclear area and shape indicate cell stress/damage [26, 27]. We investigated the nuclear morphology of Smi32+ pyramidal neurons of layers V–VI co-labelled with anti-C3b-iC3b using high-resolution confocal z-stacks (captured under sequential scanning of the blue, green and red channels, using a plan apochromat ×63/1.40 oil immersion objective, image scaling = 0.07 μm/pixel, optical section = 0.5 μm). Images of single (Smi32+, C3b−)- or double (Smi32+/C3b+)-stained cells were imported to ImageJ, and optical sections, where the nucleus was sectioned most centrally (visible nucleolus and z-section where nucleus at its widest diameter), were outlined using the “wand” tool and morphometric parameters calculated for each nucleus using the “shape descriptors” tool. A minimum of twenty co-labelled Smi32/C3b-iC3b+ neurons per case, from eight MS cases, were assessed.

### In situ hybridisation

In situ hybridisation was performed according to the method described by Budde et al. [28]. Sections previously immunostained with anti-NeuN (Additional file 1: Table S2) and detected with a goat anti-mouse-alkaline phosphatase antibody (AP, Dako, Glostrup, Denmark), visualised with Vector Blue AP substrate kit (Vector Laboratories). The hybridisation was performed using a 5′ fluorescein (FAM)-labelled 19mer antisense oligonucleotide that contains locked nucleic acid (LNA) and 2′-O-methyl (2′-O-Me)-RNA moieties (C1q: FAM-TggTccTugAugTuuCcuG, capitals indicate LNA and lower case are 2′-O-Me RNA; Ribo Task ApS, Odense, Denmark). Briefly, sections were pre-hybridised in hybridisation mix (4 M urea in 600 mM NaCl, 10 mM HEPES buffer, pH 7.5, 1 mM EDTA and ×5 Denhardt’s reagent) before probe hybridisation at 55 °C for 45 min in the same solution. Following hybridisation, sections were washed in saline-sodium citrate buffer, and the probe was detected using a sheep anti-fluorescein-AP Fab fragment (Roche Diagnostics GmbH, Penzberg, Germany) and a rabbit anti-sheep immunoglobulin G/horseradish peroxidase (HRP) (Dako). HRP was visualised using Vector NovaRED (red-brown reaction product) prior to permanent mounting (VectaMount, Vector Laboratories).

### Data presentation and statistical testing

Data was handled in Excel (Microsoft Office, 2010) and analysed using GraphPad Prism (v6.05, GraphPad Software, CA, USA). Appropriate multi-group comparisons and correlation analysis were performed following D’Agostino and Pearson normality testing. In all instances, case means per region of interest (e.g. GML, WML) were compared and a *p* < 0.05 was regarded as significant.

## Results

### Complement is activated in MS cortical and deep grey matter

Chronic inflammatory demyelinating lesions of the neocortex, thalamus and hippocampus grey matter (GM) were identified by histological and immunohistochemical assessment (Fig. 1a–d) as described in the methods. In situ hybridisation for complement *C1QA* transcript showed that complement C1q is synthesised by neurons of the deep cortical laminae in MS (Fig. 1e). Neurons and glia were immunostained with an antibody to the central complement component C3b (and its initial cleavage product iC3b) (Fig. 1f). Quantification of C3b immunopositive cells revealed an increased number in MS GMLs (of cortical and deep grey matter—thalamus), in comparison to non-neurological controls (Fig. 1g). There was an increase in the proportion of C3b+ cells with a neuronal morphology out of the total number of C3b+ quantified cells in MS cortical GM (normal appearing grey matter (GMN) and GML), in comparison to non-neurological control samples (Fig. 1h). In addition to notable labelling of cells resembling neurons (arrows in Fig. 1f), C3b immunoreactive myelin was present, frequently closely apposed with HLA-D+ phagocytes (Fig. 1i); oligodendrocytes (Olig-1+; Fig. 1j) and microglia (Iba-1+; Fig. 1k). The number of C3b immunostained cells was not associated with confounding variables such as age of death, post-mortem delay or whether death was infection related (Additional file 1: Table S3).

### Classical, alternative and terminal pathway activation products are present in MS cortical grey matter lesions

We focussed our attention on describing complement activation in association with neocortical demyelination and neurodegeneration in progressive MS. Cortical GMLs were described as subpial, leukocortical or spanning the entire cortical ribbon but without affecting the underlying white matter (Fig. 2a–d). The majority of cortical GMLs were chronic inflammatory demyelinating lesions whilst classically active cortical GMLs were infrequently observed (Fig. 2e). In a classically active cortical GML (confluent with HLA-D+ macrophages containing early myelin degradation products; Fig. 2e′–e′′), complement C1q and complement activation fragment C3b+ cells were noted (Fig. 2e′′′). We stained and quantified the density of C1q, fragment Bb and C9neo immunopositive cells in MS normal-appearing GM and chronic GML areas, in comparison to non-MS inflammatory controls and non-neurological controls. Cells with a neuronal, oligodendrocyte and/or astrocyte-like morphology were labelled by antibodies against C1q, fragment Bb and C9neo in grey and white matter areas (Fig. 2f–h; (Additional file 1: Figures S2, S3)). The pattern of cell-associated complement labelling in MS and control brain was similar to that seen in Alzheimer’s disease cortex, which was used as a positive staining control (see Additional file 1: Figure S4). Immunostaining revealed a significantly greater number of complement-labelled cells (neurons and glia) in deeper cortical laminae of the MS GMLs (leukocortical and type IV) in comparison to region and neuronal layer-matched controls (Fig. 2i–k). The number of C1q+ and C3b+ cells was elevated in active cortical GMLs (albeit with an *n* = 2) in comparison to chronic cortical GMLs (*n* = 18; 12.0 and 31.1 positive cells per field of view compared with 7.7 ± 1.8 and 12.5 ± 2.6 C1q and C3b+ cells in active and chronic GML groups, respectively). The density of C9neo+ cells, determined from unfixed, cryopreserved samples from the same cortical regions from the same MS cases, was significantly elevated in deep cortical (leukocortical and type IV) and subpial (type III) GMLs in comparison to non-inflammatory controls. WMLs generally displayed two- to tenfold more complement immunopositive cells in comparison to grey matter areas in the same tissue block. The density of C1q-, Bb- and C9neo-positive cells in MS WMLs was increased in comparison to normal-appearing and control white matter (Fig. 2i–k, Additional file 1: Figure S3).

### The expression of key complement classical, alternative and terminal pathway regulators in cortical grey matter lesions is similar in MS and controls

**Fig. 3:**
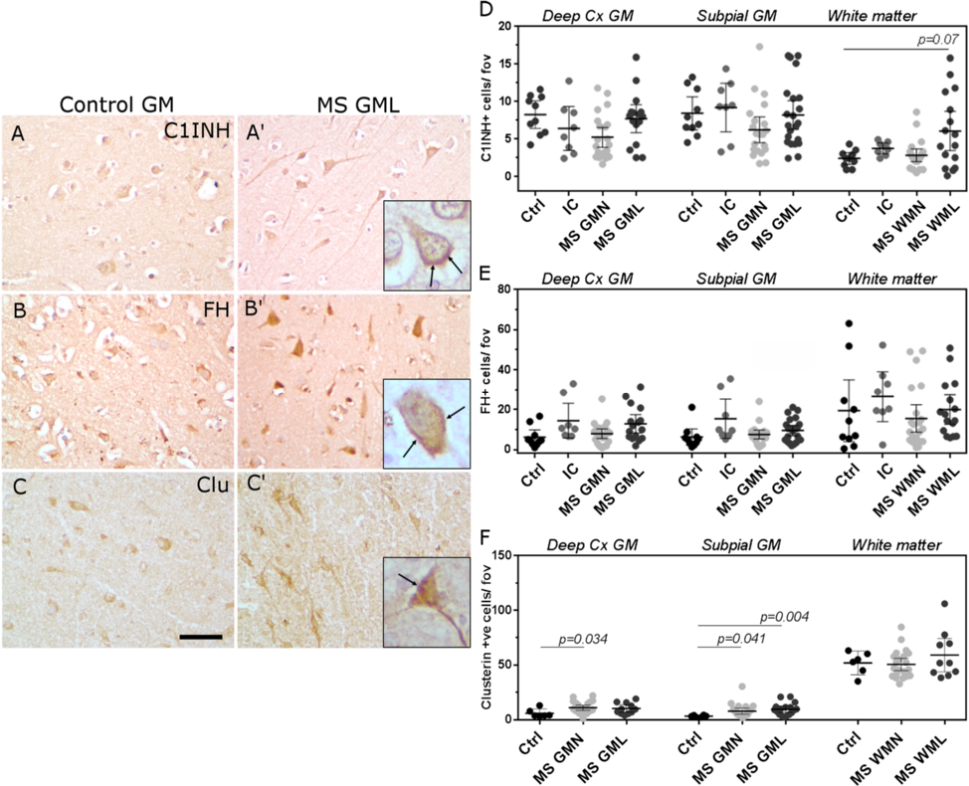
The expression of key regulators of the classical and alternative complement pathways are unchanged in MS cortical grey matter. Complement C1-inhibitor+ (C1INH, classical pathway regulator), factor H+ (FH; alternative pathway regulator) and clusterin (Clu, terminal pathway regulator) expression in and on cells of the cortical grey matter (**a**–**c**′; cells with a distinct neuronal morphology are shown in *insets* and *arrows* indicate cytoplasm/membrane immunoreactivity). The number of C1INH+ (**d**) and FH+ (**e**) cells were unchanged in GMLs in comparison to controls. There was an increase in the density of Clu+ cells (**f**) in the GMN of the deep cortical laminae and in the GMN and GMLs of the subpia. Each data point represents the mean value per area of interest and group means and 95 % confidence interval are shown. Groups compared with Kruskal-Wallis and Dunn’s multiple comparison post-test. *Scale bars*: **a**–**c**, 100 μm

We next determined the numerical density of cells immunolabelled for the classical pathway Creg, C1-inhibitor (C1INH), the alternative pathway regulator factor H (FH) and the terminal pathway regulator clusterin (Fig. 3). In agreement with our description of complement activation fragment staining, Creg staining was seen on and within cells with a neuronal and/or glial morphology in cortical grey matter areas (Fig. 3a–c, Additional file 1: Figures S2, S3). The number of C1INH and FH immunolabelled cells was unchanged between MS and control (Fig. 3d, e), whilst the density of clusterin immunostained cells was increased in MS GMN regions in comparison to non-neurological controls (Fig. 3f, note that cryosections of inflammatory controls were not available). These data imply that classical and alternative pathways are active despite unchanged expression of key Cregs in lesions of the deeper cortical laminae.

### Microglia are complement anaphylatoxin receptor positive and are increased in density in cortical grey matter lesions

**Fig. 4:**
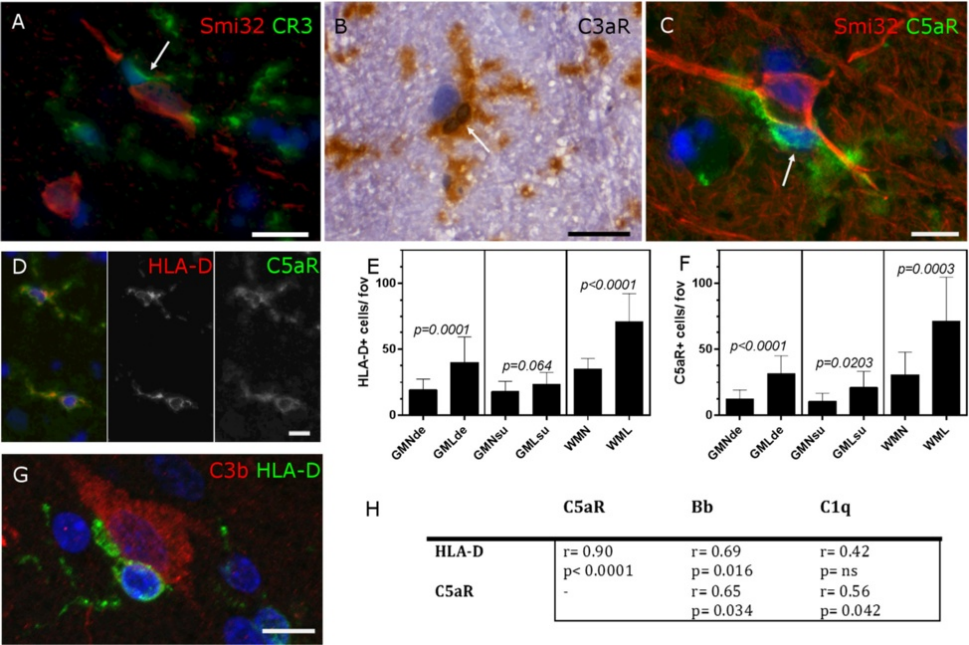
Microglial activation and complement anaphylatoxin receptor expression in the MS cortex. Expression of the opsonin receptor (CR3/CD11b) and anaphylatoxin receptors C3aR and C5aR identified microglia (*arrows* in **a**–**c**) in the MS grey matter, some of which closely apposed neurons (identified by Smi32 immunoreactivity or haematoxylin counterstain) in the demyelinated cortex (**a**–**c**). C5aR expression identified activated microglia (**d**) and the density of HLA-D+ and C5aR+ microglia was elevated in MS cortical lesions in comparison to normal-appearing grey matter (**e**, **f**). Example of an activated microglial cell associated with a C3b+ neuron (**g**). The number of HLA-D+ and C5aR+ cells were significantly correlated and the number of C5aR+ cells associated with activation of the classical and alternative complement pathways (**h**). **e**, **f**, Bar graphs of group means ± standard deviations; Mann-Whitney test. **h**, Spearman non-parametric comparison of the density of immunopositive cells in the demyelinated MS cortex. *Scale bars*: **a**, **b**, 20 μm; **c**, **d**, **g**, 10 μm

We immunostained cryopreserved MS tissue from our complete MS cohort to reveal the presence of complement receptor 3+ (CR3/CD11b), C3aR+ and C5aR+ cells with a microglial morphology in close contact with cortical neurons (Fig. 4a–c). C5aR+ microglia were activated (HLA-D+, Fig. 4d) and were significantly increased in number in GMLs and WMLs, in comparison to normal appearing tissues in the same blocks from the same cases (Fig. 4e, f). HLA-D+ microglia were seen tightly associated with C3b+ cortical neurons and the density of activated microglia (C5aR+ and HLA-D+ cells) correlated with the number of classical (C1q+) and alternative (fragment Bb+) cells in GMLs affecting the deep cortical laminae (Fig. 4h).

### Complement is associated with morphological and immunophenotypic markers of stress and neurodegeneration in MS cortical grey matter lesions

**Fig. 5:**
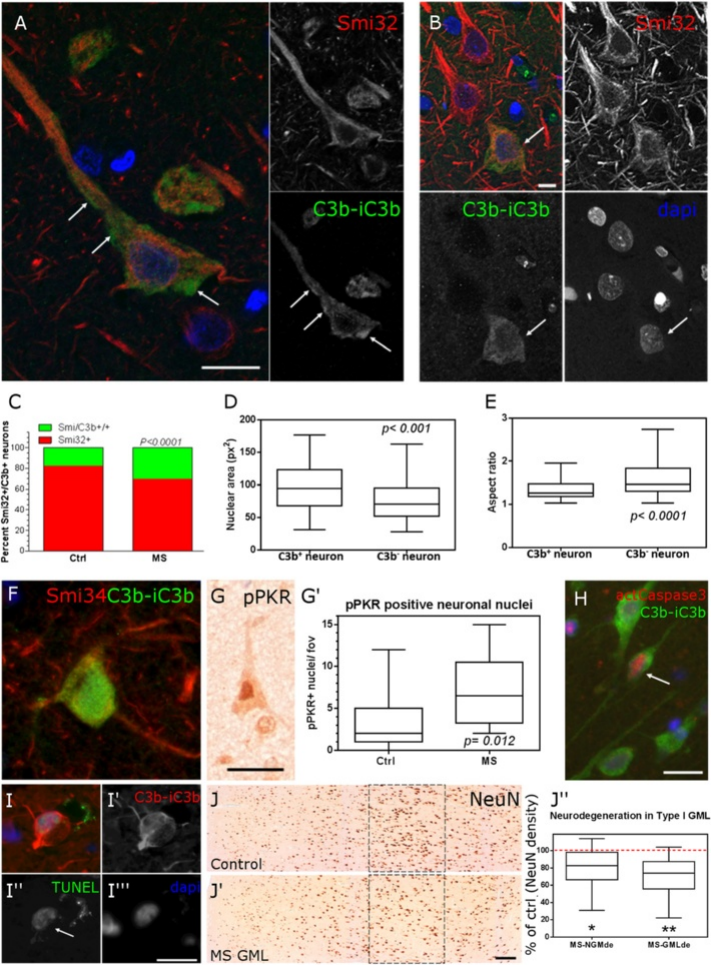
Complement-associated neuronal damage and loss. Large pyramidal neurons (Smi32+) of the deeper laminae of the demyelinated MS cortex displayed surface and cytoplasmic immunoreactive for C3b (**a**, *arrows* indicate surface-associated immunoreactivity visualised in a single 0.5-μm optical section acquired by confocal microscopy), some of which (**b**, projected z-stack image) displayed a dysmorphic nucleus (*arrow*). Twice as many neurons were immunolabelled with anti-C3b in the MS cortex in comparison to control tissues (**c**) and C3b+ neurons in MS (Smi32+) displayed a markedly altered nuclear area and aspect ratio (**d**, **e**; *Box* and *whiskers* plot of median, interquartile and minimum to maximum data range). C3b+ neurons expressed aberrant neurofilaments in the perikarya (**f**; hypo-phosphorylated neurofilaments, Smi34+) were positive for the pro-apoptotic kinase phosphorylated PKR (**g**) and the numbers of pPKR+ neuronal nuclei were elevated in MS cortex (**g**′) in comparison to control. Complement-labelled neurons occasionally expressed the apoptosis-associated markers (**h**) activated caspase3 (p17 subunit) and (**i**–**i**′′′) DNA strand breaks (TUNEL reaction positive, C3b+ neuron). The density of NeuN+ neurons was reduced in normal appearing (18 % reduction) and lesioned (25 % reduction) layer V grey matter, in comparison to non-neurological controls (**j**–**j**′′). Group medians compared by the non-parametric Mann-Whitney test (**c**–**e**) or Kruskal-Wallis and Dunn’s multiple comparison post-test (**j**′′). *Scale bars*: **a**, **b**, 50 μm; **f**–**i**, 25 μm; **j**, **j**′, 200 μm

C3b+ neurons of the deeper cortical laminae were identified by their co-expression of non-phosphorylated neurofilament protein (Smi32+, Fig. 5a, b). Complement activation fragment C3b immunostaining visualised by confocal microscopy was noted on the surface and in the cytoplasm of cortical neurons (Fig. 5a), whilst some of these cells had an altered nuclear morphology (Fig. 5b). The proportion of cortical neurons (Smi32+) that were complement C3b+ was increased in MS GML (Fig. 5c). C3b+ neurons in MS cortex showed signs of nuclear stress as defined by morphometric analysis of nuclear area and shape (aspect ratio; Fig. 5d, e) [27]. Quantitative evidence of neuronal dysmorphia was supported by the presence of C3b+ neurons expressing hypo-phosphorylated neurofilaments (Smi34+), neuronal nuclei expressing the pro-apoptotic kinase and neuronal stress/damage marker protein kinase R (pPKR) [29]—that were more numerous in MS GM in comparison to control GM (Fig. 5g, g′), and the presence of C3b+/cleaved caspase3+ or C3b+/TUNEL reaction+ cortical neurons (Fig. 5h, i). Within the same regions of interest, there was a significant reduction in the number of NeuN+ neurons in normal (*p* = 0.013) and lesioned cortical layer V MS GM (*p* = 0.002) in comparison to region matched non-neurological controls (Fig. 5j–j′′).

## Discussion

Progressive MS is associated with a widespread and chronic activation of the central immune response confined behind a relatively intact blood-brain barrier [2, 30]. Our quantitative analysis demonstrates that complement classical, alternative and terminal pathways are activated and we show for the first time that complement expression is notable in and on large neurons in MS cortical grey matter lesions. Our data suggest that a dysregulation of complement activation and control occurs in the MS brain, and an increase in complement anaphylatoxin receptor-positive microglia may serve to sustain the neuroinflammatory response that drives myelin and neuronal pathology in the progressive phase.

### Complement is activated in MS cortical grey matter lesions

In the cortical grey matter, C1q expression was seen in and on neurons, neurites and glia. The pattern of staining suggested biosynthesis as well as a deposition of complement, which is supported by *C1q* mRNA expression in NeuN+ neurons, shown here and by others [22]. Evidence for alternative complement pathway activation in our study is seen in the elevated number of activation fragment Bb+ cells. Therefore, localisation of the opsonin C3b (and its breakdown product iC3b) in and on neurons in GMLs of the cortex, thalamus and hippocampus may be a consequence of classical and/or alternative pathway activation.

Proteolysis of C3 yields C3a, a soluble anaphylatoxin that activates both protective and damaging immune responses against neurons through C3aR engagement. Membrane bound C3b activates CR3+ microglia to trigger activation that can be detrimental to cell integrity, whilst recent evidence suggests that C3a and C3b can be generated intracellularly [31]. Accumulation of C3b can lead to formation of the C5 convertase and release of C5a, which is damaging to neurons via C5aR activation [32, 33]. We show that the number of C5aR+ microglia is increased in chronic cortical lesions. C5b formation and the subsequent recruitment of C6-9 lead to the formation of MAC. MAC deposition is seen in acute and chronic MS WMLs [19, 34, 35] but hitherto has not been demonstrated for cortical GMLs. The MAC may be directly cytopathic (there is a significant loss of cortical neurons in these same lesions of interest, Fig. 5), but even sublethal attack can trigger the production of pro-inflammatory cytokines and reactive oxygen species, stimulate binding of damage-associated molecular patterns [36], confer protection [37] or mediate NLRP3 inflammasome-induced IL1β synthesis [38].

Our quantitative findings are in agreement with qualitative reports describing C1q, C3d and C4d immunoreactivity in some cortical GMLs [18–21], and quantitative measures of elevated C1q and C3d labelling in the hippocampus [22], in a cell-associated pattern of immunostaining similar to that seen by us in the cortical grey matter. In agreement with previous publications [18, 19], we noted that the pattern of complement immunoreactivity (recognition molecules and activation fragments) was most striking in cortical layers V–VI near the white matter border; however, we report that the density of complement-labelled cells was not related to the presence of underlying WMLs (Additional file 1: Figure S5), confirming that a major part of the complement response is generated locally by cells of the cortical grey matter.

### Complement regulator expression in MS cortical grey matter lesions does not increase with activation—evidence for dysregulation?

Uncontrolled activation of complement is detrimental to the host and results in progressive cell and tissue injury in the chronically inflamed organ [13]. Each complement activation pathway is regulated at multiple strategic points to prevent uncontrolled activation. We did not detect a significant difference in the numerical density of cells immunolabelled for C1INH or FH between MS cortical grey matter and control samples. The demonstration of unchanged number of complement regulator-positive cells despite on-going complement activation suggests that the drive to complement activation overwhelms regulation. We suggest that such a disparity between markers of activation and regulation of the classical and alternative complement pathways manifests as the robust and widespread expression of fragments of complement activation, leading to the unchecked generation of opsonin and soluble anaphylatoxin products that may exacerbate the pathology in the progressive MS brain. It is for these reasons that complement markers could be valuable prognostic indicators of a more severe disease [15, 17].

### Microglial activation and neuronal injury and loss

We are interested in the pathomechanisms of cortical and neuronal injury due to their relevance to disability progression in MS [1]. There was significant loss of neurons from the deep cortical laminae that corresponded to the areas of elevated numbers of complement-labelled cells and a greater proportion of large neurons co-labelled with C3b. Loss of cortical neurons will be a consequence of numerous factors, including demyelination, cytokine or cell-mediated cytotoxicity, de-innervation, retrograde degeneration, mitochondrial dysfunction and excitotoxicity (reviewed by [39]), to which biosynthesis and deposition of products of complement activation, in the presence of increased numbers of complement receptor positive microglia, could be contributory. These findings suggest that in this environment of significant neuronal loss, a substantial proportion of the remaining large (Smi32+) neurons are under inflammatory stress and appear dysmorphic, which would render them dysfunctional. Complement may drive neuronal damage through C3b(iC3b)-CR3 activation of phagocytes in a process of primary phagocytic damage [40] or through the activation of local glia through anaphylatoxin receptor engagement that can cause dendritic damage and neuronal toxicity [10, 41, 42]. Complement synthesised by neurons could be a physiological response to stress, which may aid synaptic pruning by locally activated microglia or engage neuronal complement receptors resulting in altered neuritic and synaptic presentation [9, 22].

## Conclusions

The presence of complement activation products and anaphylatoxin receptor positive activated microglia suggest that neurons in the MS cortical grey matter are subject to a sustained innate immune attack that may contribute to their dysfunction and death. Our work supports efforts to investigate the utility of complement as a potential biomarker or therapeutic target for progressive MS.

## Abbreviations

C1INH, C1 inhibitor; Clu, clusterin; Creg, complement regulator; Ctrl, control cohort; FH, factor H; GML, grey matter lesion; GMN, normal appearing grey matter; IC, non-MS inflammatory controls; MAC, membrane attack complex; MOG, myelin-oligodendrocyte glycoprotein; WML, white matter lesion

## Additional file

Additional file 1: Table S1. Demographic and pathological details for cases used in this study. All cases were retrospectively confirmed as MS, non-inflammatory or inflammatory (viral encephalitis or ischaemia) following detailed analysis of patient health records and full neuropathological work up. Region matched sections (FFPE or cryosections) were available for all cases from the frontal cortex (sup. Frontal gyrus), cingulate cortex and hippocampus and temporal (inf.) gyrus. Tissue sections from inflammatory control cases were selected based on region-matching and not on the presence of pathology in the particular block. Examples of myelin and inflammatory cell staining are shown in Additional file: **Figure S1.** Abbreviations:  PMD- post-mortem delay from death to tissue processing (hrs); Disease activity- neuropathologically defined disease activity based on the presence (Active) or absence (Stable) of active inflammatory lesions; Disease duration is calculated from retrospectively determined time of disease onset; Tissue- available formalin-fixed paraffin embedded (FFPE) or cryopreserved tissue blocks; Quantified lesions- number of GM or WM lesion regions of interest analysed in this quantitative study. **Table S2.** Primary antibodies used in this study, retrieval conditions and commercial source. Citrate- 100mM sodium citrate, 10mM citric acid, pH 6.0; TRIS/ EDTA- 10mM Tris, 1mM EDTA, pH 8.5. **Table S3.** Complement cell-associated expression was not related to age of death, post-mortem delay or whether death was infection (inflammatory) or non-infection related. Linear regression and Pearson’s correlation analysis (Age of death and PMD (post mortem delay) versus mean C3b+ cell count per case) or Mann-Whitney test of mean C3b+ cell count between those cases with an inflammatory-related or non-inflammatory disease noted at death. **Figure S1.** Non-neurological controls and inflammatory neurological controls used in this study. Control cortical grey and subcortical white matter was characterised by a uniform pattern of anti-MOG staining (A- D) and small numbers of ramified HLA-D+ microglia (E- H). Inflammatory controls were selected on the basis of available region-matched blocks and frequently displayed an unremarkable pathology. Occasional areas of microglial activation were noted in some sections of cortical and subcortical tissue (I- P; ischaemic encephalitis inflammatory control), whilst perivascular cuffs of activated microglia and macrophages were sometimes seen in cases of viral encephalitis (Q- X). Scale bars= W (and all low power images) 100µm; X (and all high power images) 20µm. **Figure S2.** Complement recognition fragment, activation products and regulator expression in MS, non-MS inflammatory controls (IC) and non-neurological control (Ctrl) grey matter. 400x magnification images of C1q (A-C), C1-inhibitor (D-F), Bb (G-I), factor H (J-L), C3b-iC3b (M-O), MAC (P, Q) and clusterin (R, S) in deeper cortical laminae of control (Ctrl; left column & P, R), MS (central column & Q, S) and non-MS inflammatory controls (right column only) showing the cell-specific and often robust pattern of anti-complement immunostaining seen in MS (and to a lesser extent, non-MS inflammatory controls) cortical grey matter in comparison to non-diseased controls. Scale bar= 20µm. **Figure S3.** Complement recognition fragment, activation product and regulator expression in subcortical white matter samples. 400x magnification images of C1q (A-C), C1-inhibitor (D-F), Bb (G-I), factor H (J-L), C3b-iC3b (M-O), MAC (P, Q) and clusterin (R, S) in sub-cortical white matter of non-neurological control (Ctrl), MS and non-MS inflammatory controls (IC), showing the cell-specific and often robust pattern of anti-complement immunostaining seen in MS white matter in comparison to non-neurological controls. Scale bar= 20µm. **Figure S4.** Examples of complement immunolabelling in Alzheimer’s disease (AD). Two AD cases (age of death, 85 and 96yrs; gender, both female; post-mortem delay, 30 and 19hrs, respectively), showed complement specific immunolabelling with anti-C1q, C3b-iC3b and C9neo of cells resembling neurons and glia (similar to that seen in MS and controls), as well as diffuse labelling of amyloid plaques (for anti-C1q, C3b and C9neo antibodies). Cellular immunolabelling of C1INH and clusterin resembles that seen in control, MS and inflammatory control cases. C5aR+ glia were also seen in the AD deep grey matter. All images captured at 200x magnification. **Figure S5.** Complement activation in cortical grey matter is not related to underlying white matter inflammation. Grey matter lesions of the deeper cortical laminae are characterised as type I (involving both the white and grey matter) or type IV (involving the entire vertical extent of the cortex but without affecting the underlying white matter). Our analysis revealed there to be no difference between the density of C1q (A,B and D) and C3b-labelled cells (C) in type I grey matter lesions (that are associated with white matter lesions) and type IV lesions. Scatter graphs of type I versus type IV cell-specific counts for C1q and C3b quantitative immunostaining compared by Mann-Whitney test. (PDF 1849 kb)
