# Supplementary material for: Complement is activated in progressive multiple sclerosis cortical grey matter lesions
Source: J Neuroinflammation. 2016 Jun 22;13:161. doi: 10.1186/s12974-016-0611-x (PMC4918026; doi:10.1186/s12974-016-0611-x)
Supplement: Additional file 1: Table S1. — Demographic and pathological details for cases used in this study. All cases were retrospectively confirmed as MS, non-inflammatory or inflammatory (viral encephalitis or ischaemia) following detailed analysis of patient health records and full neuropathological work up. Region matched sections (FFPE or cryosections) were available for all cases from the frontal cortex (sup. Frontal gyrus), cingulate cortex and hippocampus and temporal (inf.) gyrus. Tissue sections from inflammatory control cases were selected based on region-matching and not on the presence of pathology in the particular block. Examples of myelin and inflammatory cell staining are shown in Additional file: Figure S1. Abbreviations: PMD- post-mortem delay from death to tissue processing (hrs); Disease activity- neuropathologically defined disease activity based on the presence (Active) or absence (Stable) of active inflammatory lesions; Disease duration is calculated from retrospectively determined time of disease onset; Tissue- available formalin-fixed paraffin embedded (FFPE) or cryopreserved tissue blocks; Quantified lesions- number of GM or WM lesion regions of interest analysed in this quantitative study. Table S2. Primary antibodies used in this study, retrieval conditions and commercial source. Citrate- 100mM sodium citrate, 10mM citric acid, pH 6.0; TRIS/ EDTA- 10mM Tris, 1mM EDTA, pH 8.5. Table S3. Complement cell-associated expression was not related to age of death, post-mortem delay or whether death was infection (inflammatory) or non-infection related. Linear regression and Pearson’s correlation analysis (Age of death and PMD (post mortem delay) versus mean C3b+ cell count per case) or Mann-Whitney test of mean C3b+ cell count between those cases with an inflammatory-related or non-inflammatory disease noted at death. Figure S1. Non-neurological controls and inflammatory neurological controls used in this study. Control cortical grey and subcortical white matter was characterised by a u [file 12974_2016_611_MOESM1_ESM.pdf]

Supplementary Table 1

Case	Sex	Age of Death	Cause of Death	PMD (hrs)	MS Type	Disease Activity	Disease Duration (yrs)	Tissue		Quantified Lesions			
										FFPE		Frozen	
								FFPE	Frozen	GM	WM	GM	WM
MS Cohort for Quantification													
402	M	46	MS, Broncopneumonia	12	SPMS	Stable	20	Y	Y	3	2	1	1
405	M	62	MS, Septicaemia, Metastatic Colon Cancer	12	SPMS	Active	25	Y	Y	3	1	1	1
407	F	44	Septicaemia, Pneumonia	22	SPMS	Active	19	Y	Y	5	1	2	1
408	M	39	Pneumonia, Sepsis	21	SPMS	Active	10	Y	Y	4	1	2	1
422	M	58	Chest infection due to MS	25	SPMS	Active	-	Y	Y	4	1	2	1
423	F	54	Pneumonia	11	SPMS	Active	30	Y	Y	4	1	2	1
425	F	46	Pneumonia, MS	25	SPMS	Active	21	Y	Y	3	1	2	1
438	F	53	MS	17	SPMS	Active	18	Y	Y	4	1	2	1
444	M	49	Renal Failure	18	SPMS	Stable	20	Y	Y	2			
473	F	39	Broncopneumonia, MS	9	PPMS	Active	13	Y	Y	1			
485	F	57	Broncopneumonia, Advanced MS	24	PPMS	Active	29	Y	Y	2	1	2	
491	F	64	Anaphylactic Reaction	9	SPMS	Active	26	Y	Y	1	1		
492	F	66	Sigmoid Cancer	15	PPMS	Active	31	Y	Y	2	1		
497	F	60	Aspiration Pneumonia, MS	26	SPMS	Active	29	Y	Y	3	1	2	
510	F	38	Pneumonia, MS	19	SPMS	Active	22	Y	Y	3	1	2	
513	M	51	MS, Respiratory Failure	17	SPMS	Active	18	Y	Y	3	1	2	1
517	F	48	Sepsis, MS	12	PPMS	Active	25	Y	Y	2	1	1	
523	F	63	Broncopneumonia, MS	20	SPMS	Stable	32	Y	Y	3	1	1	
527	M	47	Pneumonia, MS	10	SPMS	Active	25	Y	Y	2		1	1
528	F	45	MS	17	SPMS	Active	25	Y	Y	1	1		
530	M	42	MS	15	SPMS	Active	24	Y	Y	4	1	1	1
538	M	50	Pneumonia	12	SPMS	Stable	39	Y	Y	1			
N= 22	9 M<br>13 F	50<br>(38-66)		17<br>(9-26)	4 PPMS,<br>18 SPMS	4 Stable<br>18 Prog	25<br>(10-39)	22 Y	22 Y	3<br>(1-5)	1<br>(0-1)	2<br>(0-2)	1<br>(0-1)

Case	Sex	Age of Death	Cause of Death	PMD (hrs)	MS Type	Disease Activity	Disease Duration	Tissue	Quantified Lesions	
									FFPE	Frozen

Case	Sex	Age of Death	Cause of Death	PMD (hrs)	MS Type	Disease Activity	Disease Duration (yrs)	Tissue		Quantified Lesions			
										FFPE		Frozen	
								FFPE	Frozen	GM	WM	GM	WM
							(yrs)						
Non-neurological Control Cohort for Quantification													
CO25	M	35	Carcinoma of the Tongue	22				Y	Y				
CO26	F	78	Myeloid Leukaemia	33				N	Y				
CO32	M	88	Prostate Cancer, Bone Metastases	22				N	Y				
CO37	M	84	Bladder Cancer, Pneumonia	5				N	Y				
CO45	M	77	Cardio Pulmonary Degeneration, Prostate Cancer, old age, Alzheimers	22				N	Y				
CO48	M	68	Colon Cancer	10				N	Y				
12/023	M	69	Unknown	24				Y	N				
12/046	M	72	Unknown	24				Y	N				
12/048	F	65	Ovarian Cancer	48				Y	N				
12/052	F	42	Pancreatic Cancer	48				Y	N				
12/088	M	51	Cardiac Arrest	24				Y	N				
11/093	F	52	Chronic Liver Disease	48				Y	N				
11/122	F	65	Unknown	24				Y	N				
12/132	F	67	Unknown	48				Y	N				
1231/93	M	58	Unknown	n/a				Y	N				
N= 15	9 M<br>6 F	67<br>(35-88)		24<br>(5-48)				10 Y<br>5 N	6 Y<br>9 N				

Case	Sex	Age of	Cause of Death	PMD	MS Type	Disease	Disease	Tissue	Quantified Lesions

Case	Sex	Age of Death	Cause of Death	PMD (hrs)	MS Type	Disease Activity	Disease Duration (yrs)	Tissue		Quantified Lesions			
										FFPE		Frozen	
								FFPE	Frozen	GM	WM	GM	WM
		Death		(hrs)		Activity	Duration (yrs)			FFPE		Frozen	
								FFPE	Frozen	GM	WM	GM	WM
Non-MS inflammatory Control Cohort for Quantification													
B4938	M	18	HSE	n/a				Y	N				
C2342	M	17	HIV	24				Y	N				
C3727	M	41	HIV	n/a				Y	N				
C4138	M	59	CMV	n/a				Y	N				
91/1343	M	32	Broncopneumonia, Ischaemia	48				Y	N				
1140/95	F	65	Ischaemic Encephalopathy	n/a				Y	N				
1078/95	M	32	Ischaemic Encephalopathy	24				Y	N				
1062/00	F	49	Ischaemic Encephalopathy	72				Y	N				
N= 8	6 M<br>2 F	37<br>(17-65)		36<br>(24-72)				8 Y	8 N				

Supplementary Table 1 Demographic and pathological details for cases used in this study. All cases were retrospectively confirmed as MS, non-inflammatory or inflammatory (viral encephalitis or ischaemia) following detailed analysis of patient health records and full neuropathological work up. Region matched sections (FFPE or cryosections) were available for all cases from the frontal cortex (sup. Frontal gyrus), cingulate cortex and hippocampus and temporal (inf.) gyrus. Tissue sections from inflammatory control cases were selected based on region-matching and not on the presence of pathology in the particular block. Examples of myelin and inflammatory cell staining are shown in supplementary Figure 1. Abbreviations: PMD- post-mortem delay from death to tissue processing (hrs); Disease activity- neuropathologically defined disease activity based on the presence (Active) or absence (Stable) of active inflammatory lesions; Disease duration is calculated from retrospectively determined time of disease onset; Tissue- available formalin-fixed paraffin embedded (FFPE) or cryopreserved tissue blocks; Quantified lesions- number of GM or WM lesion regions of interest analysed in this quantitative study.

Supplementary Table 2

Target	Antigen	Clone	Retrieval	Supplier
Cleaved Caspase-3	Activated Caspase-3	PC	Citrate	Promega
Bb neo	Bb	A252	Citrate	Pathway Diagnostics
C9 neo (TCC)	B7	B7	n/a	In-house
Clusterin	Clusterin	552886	n/a	BD Pharmingen
CR3	CD11b	5C6	n/a	ABD Serotec
C1 Inhibitor	C1 Inhibitor	PC	TRIS/EDTA	In House
C1q	C1q	A0136	TRIS/EDTA	Dako
C3a Receptor	C3aR	hC3aRZ8	n/a	Hycult Biotech
C3b/iC3b	C3b	C330	TRIS/EDTA	In-house
C4d neo	C4d	A213	TRIS/EDTA	Pathway Diagnostics
C5a Receptor	CD88	PC	n/a	BD Pharmingen
Factor H	Factor H	PC	TRIS/EDTA	In-house
Astrocytes	GFAP	PC	Citrate	Dako
Microglia/Monocytes	HLA-DR,Q,B	Cr3/43	Citrate	Dako
Myelin	MOG	Y10	TRIS/EDTA	In-house
Neurons	NeuN	A60	Citrate	Merck-Millipore
NF-H Non-P	Non-P NFil	SMI32	Citrate	Merck
NF-H	NFil	SMI34	TRIS/EDTA	Abcam
pThr451	PKR	PC	TRIS/EDTA	Life Technologies

Supplementary Table 2 Primary antibodies used in this study, retrieval conditions and commercial source. Citrate- 100mM sodium citrate, 10mM citric acid, pH 6.0; TRIS/ EDTA- 100mM Tris, 10mM EDTA, pH 8.5.

Supplementary Table 3

	Age of Death (yrs)	PMD (hrs)	Inflammation/ Non-inflammatory
**C3b**	*r = -0.1363*<br>*p = 0.4080*	*r = -0.0526*<br>*p = 0.7875*	*p = 0.1036*

Supplementary Table 3 Complement cell-associated expression was not related to age of death, post-mortem delay or whether death was infection (inflammatory) or non-infection related. Linear regression and Pearson's correlation analysis (Age of death and PMD (post mortem delay) versus mean C3b+ cell count per case) or Mann-Whitney test of mean C3b+ cell count between those cases with an inflammatory-related or non-inflammatory disease noted at death (see supplementary Table 1 for individual case details).

Supplementary Figure 1

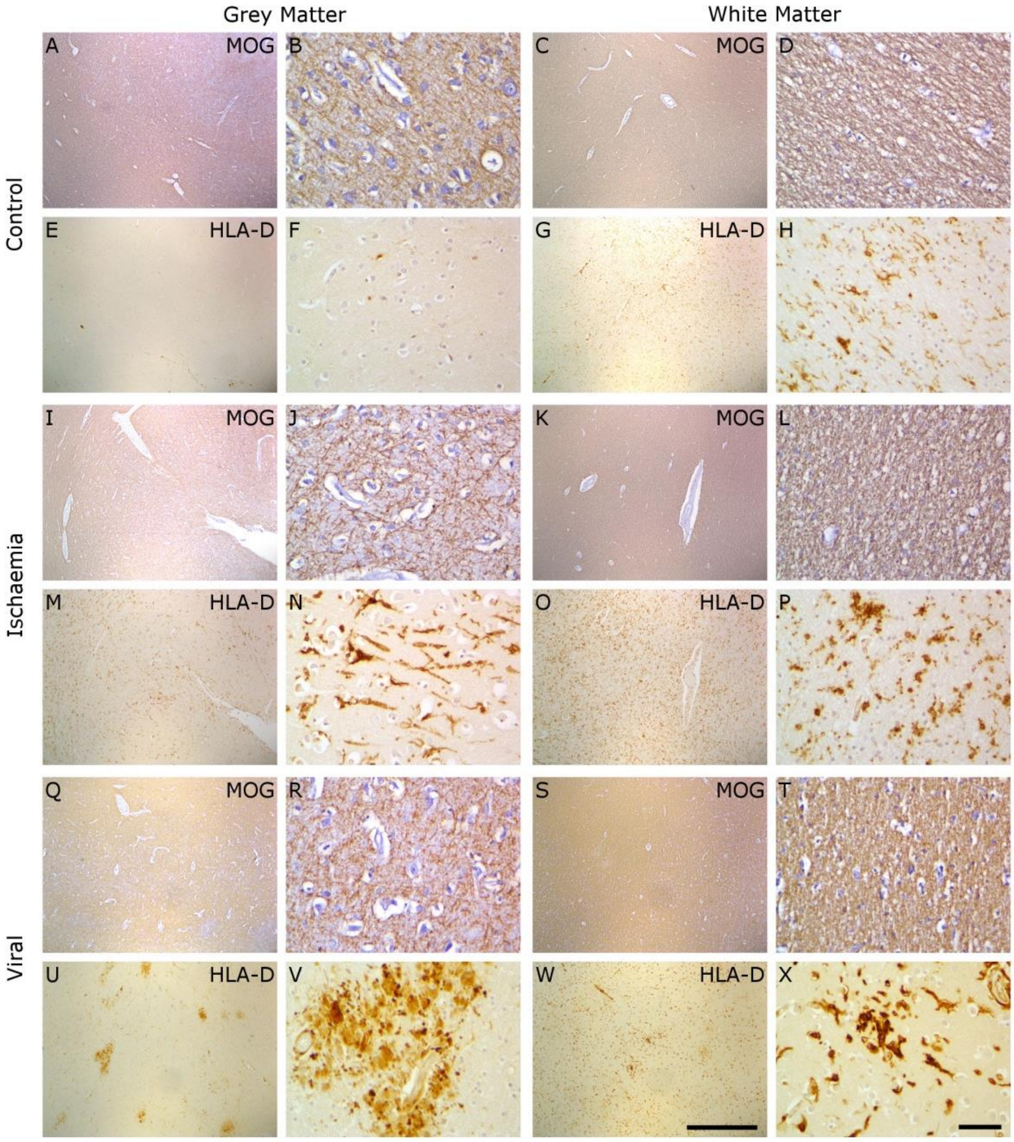

Supplementary Figure 1 Non-neurological controls and inflammatory neurological controls used in this study. Control cortical grey and subcortical white matter was characterised by a uniform pattern of anti-MOG staining (A- D) and small numbers of ramified HLA-D+ microglia (E- H). Inflammatory controls were selected on the basis of available region-matched blocks and frequently displayed an unremarkable pathology. Occasional areas of microglial activation were noted in some sections of cortical and subcortical tissue (I- P; ischaemic encephalitis inflammatory control), whilst perivascular cuffs of activated microglia and macrophages were sometimes seen in cases of viral encephalitis (Q-X). Scale bars= W (and all low power images) 100µm; X (and all high power images) 20µm.

Supplementary Figure 2

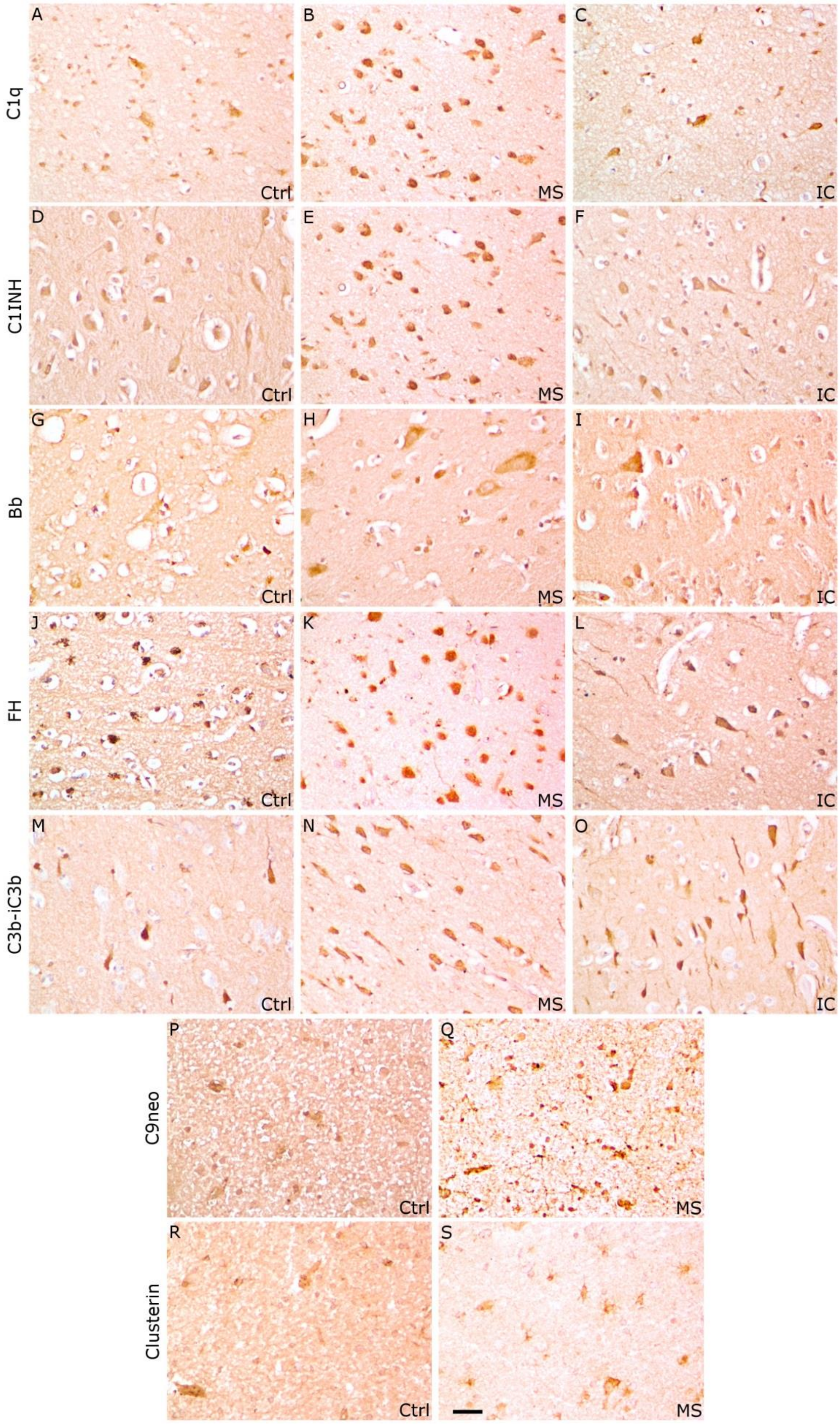

Supplementary Figure 2 Complement recognition fragment, activation products and regulator expression in MS, non-MS inflammatory controls (IC) and non-neurological control (Ctrl) grey matter. 400x magnification images of C1q (a-c), C1-inhibitor (D-F), Bb (G-I), factor H (J-L), C3b-iC3b (M-O), MAC (P, Q) and clusterin (R, S) in deeper cortical laminae of control (Ctrl; left column & P, R), MS (central column & Q, S) and non-MS inflammatory controls (right column only) showing the cell-specific and often robust pattern of anti-complement immunostaning seen in MS (and to a lesser extent, non-MS inflammatory controls) cortical grey matter in comparison to non-diseased controls. Scale bar= 20µm.

Supplementary Figure 3 (next page). Complement recognition fragment, activation product and regulator expression in subcortical white matter samples. 400x magnification images of C1q (a-c), C1-inhibitor (D-F), Bb (G-I), factor H (J-L), C3b-iC3b (M-O), MAC (P, Q) and clusterin (R, S) in sub-cortical white matter of non-neurological control (Ctrl), MS and non-MS inflammatory controls (IC), showing the cell-specific and often robust pattern of anti-complement immunostaining seen in MS white matter in comparison to non-neurological controls. Scale bar= 20µm.

Supplementary Figure 3

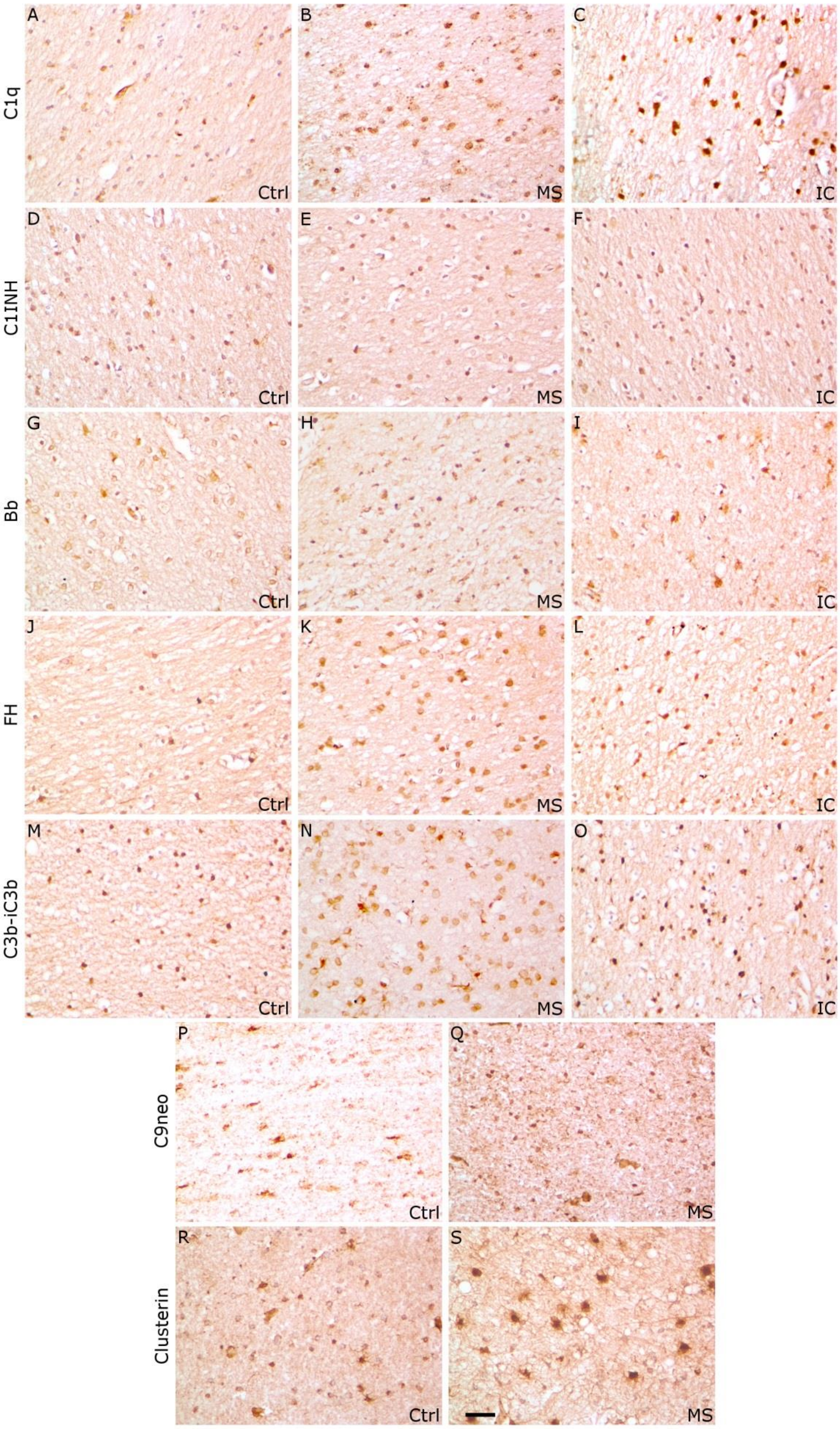

Supplementary Figure 4

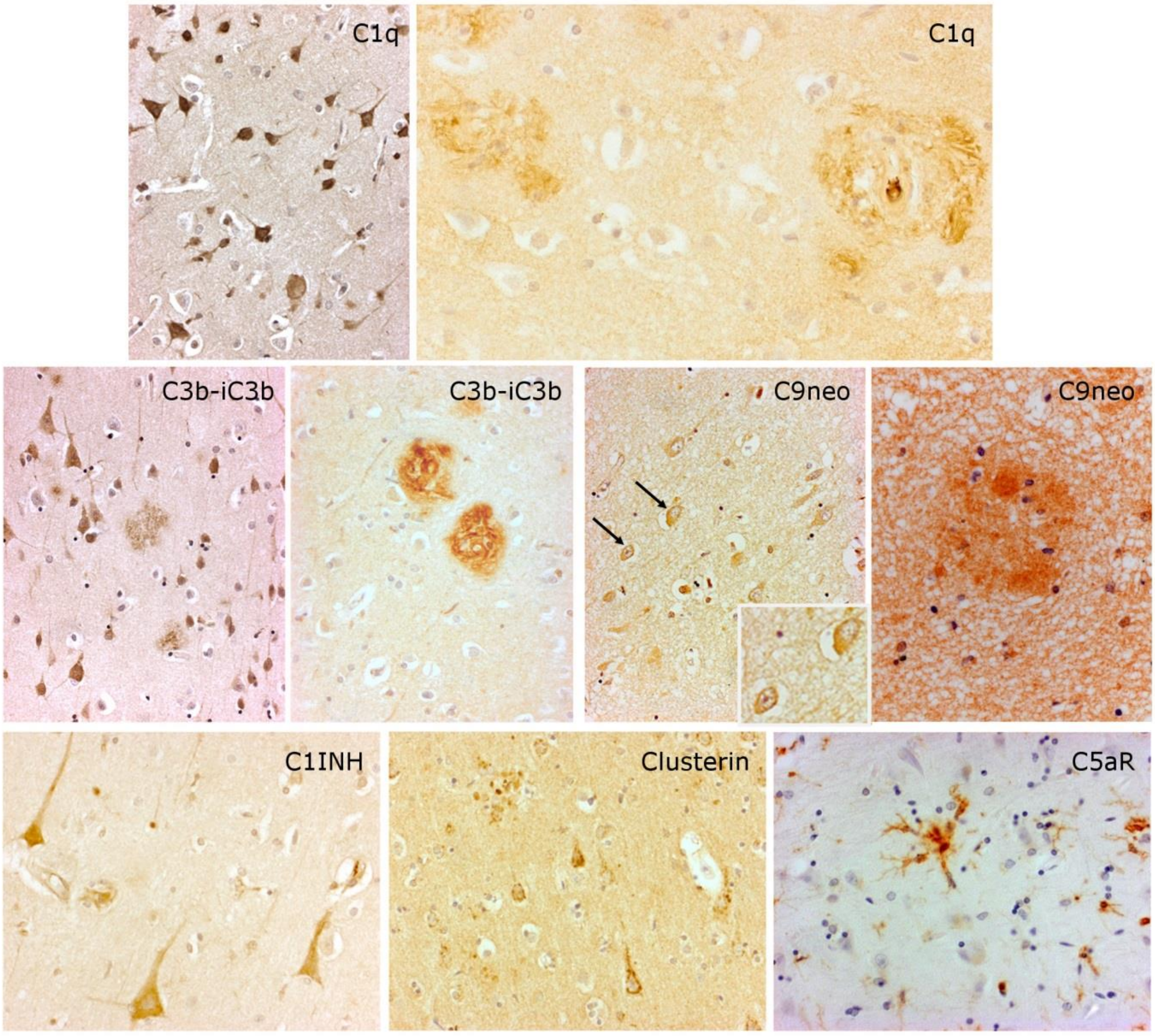

Supplementary Figure 4 Examples of complement immunolabelling in Alzheimer's disease (AD). Two AD cases (age of death, 85 and 96yrs; gender, both female; post-mortem delay, 30 and 19hrs, respectively), showed complement specific immunolabelling with anti-C1q, C3b-iC3b and C9neo of cells resembling neurons and glia (similar to that seen in MS and controls), as well as diffuse labelling of amyloid plaques (for anti-C1q, C3b and C9neo antibodies). Cellular immunolabelling of C1INH and clusterin resembles that seen in control, MS and inflammatory control cases. C5aR+ glia were also seen in the AD deep grey matter. All images captured at 200x magnification.

Supplementary Figure 5

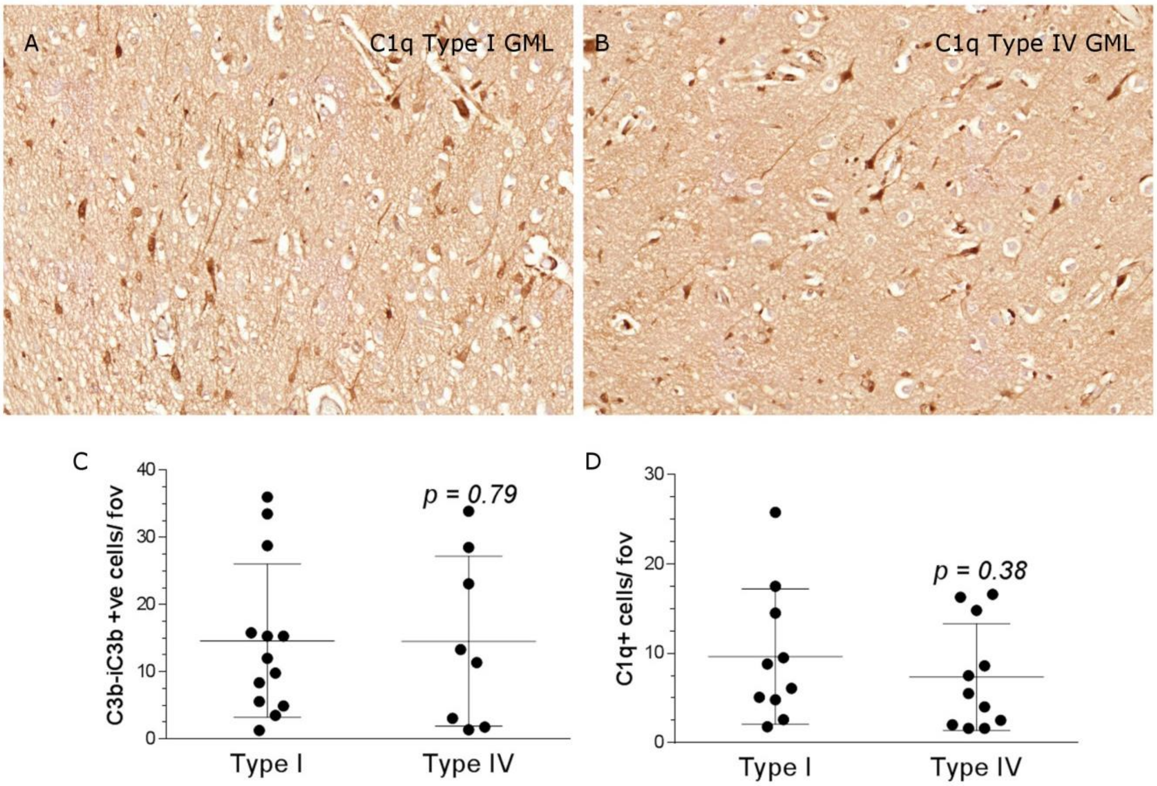

Supplementary Figure 5 Complement activation in cortical grey matter is not related to underlying white matter inflammation. Grey matter lesions of the deeper cortical laminae are characterised as type I (involving both the white and grey matter) or type IV (involving the entire vertical extent of the cortex but without affecting the underlying white matter). Our analysis revealed there to be no difference between the density of C1q (A,B and D) and C3b-labelled cells (C) in type I grey matter lesions (that are associated with white matter lesions) and type IV lesions. Scatter graphs of type I versus type IV cell-specific counts for C1q and C3b quantitative immunostaining compared by Mann-Whitney test.
